# A guanidyl-functionalized TiO_2_ nanoparticle-anchored graphene nanohybrid for enhanced capture of phosphopeptides†

**DOI:** 10.1039/c8ra05006f

**Published:** 2018-08-20

**Authors:** Hailong Liu, Bin Lian

**Affiliations:** College of Life Sciences, Nanjing Normal University No. 1, WenYuan Road, QiXia District Nanjing 210023 Jiangsu Province China bin2368@vip.163.com; State Key Laboratory of Environmental Geochemistry, Institute of Geochemistry, Chinese Academy of Sciences Guiyang China

## Abstract

TiO_2_-based MOAC (metal oxide affinity chromatography) nanomaterials are regarded as one of the most promising materials for phosphopeptide enrichment. However, the serious non-specific adsorption of acidic peptides and the limited chemisorption performance to phosphopeptides will greatly reduce the enrichment efficiency. To overcome the above problems, a novel TiO_2_ hybrid material with guanidyl-functionalized TiO_2_ nanoparticles (GF-TiO_2_) anchored on the surface of a graphene oxide (GO) platform (denoted as GF-TiO_2_–GO) is successfully synthesized and applied as a biofunctional adsorbent for selective enrichment of trace phosphopeptides. Due to the improved selectivity to phosphopeptides and larger loading capacity, the novel GF-TiO_2_–GO nanohybrids exhibited higher selectivity toward phosphopeptides and a lower detection limit even when the concentration of β-casein was decreased to only 1 × 10^−11^ M. The selective enrichment test toward phosphopeptides from the tryptic digests of nonfat milk and human serum further validated that the GF-TiO_2_–GO nanohybrids were capable of selectively capturing global phosphopeptides from complicated biological samples.

## Abstract 2

A novel TiO_2_-based MOAC hybrid nanomaterial was successfully synthesized and applied as a biofunctional adsorbent for selective enrichment of trace phosphopeptides.

## Graphical abstract

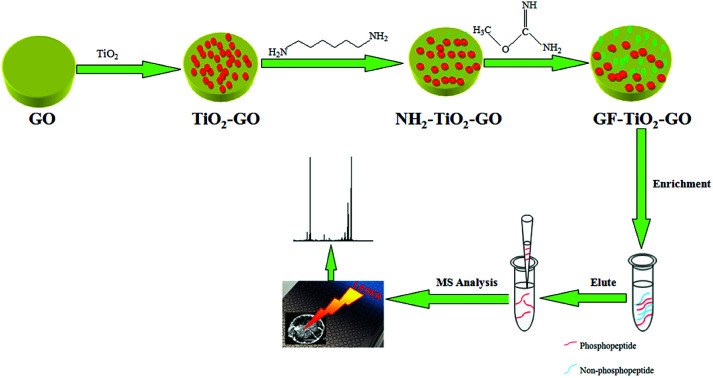

## 1. Introduction

TiO_2_-based metal oxide affinity chromatography (MOAC) nanomaterials are regarded as one of the most promising materials for phosphopeptide enrichment due to the specific chemisorption toward phosphate groups.^1–4^ However, the serious non-specific adsorption of acidic peptides greatly reduces the specificity for enrichment of phosphopeptides and the limited surface area also results in a limited chemisorption performance of phosphopeptides. Although there exists some drawbacks for the TiO_2_-based MOAC nanomaterials in the enrichment of phosphopeptides, the performance of TiO_2_-based materials toward phosphopeptide enrichment is still better than that of conventional enrichment materials.^5–8^ Therefore, it is necessary to circumvent the above problems existing in TiO_2_-based materials to develop a novel TiO_2_-based MOAC nanomaterial to selectively and sensitively enrich trace phosphopeptides in real biological samples.^9,10^

Graphene-based materials have attracted extensive research interest in recent years due to their high specific surface area and biocompatibility. These unique features have made these materials become an ideal support in the preparation of the novel graphene-based hybrid materials for the enrichment of phosphopeptides.^11–14^ Recently, it has been proved that guanidyl groups could be regarded as a better functionalized group to improve the binding of the phosphate groups and prevent the binding of other peptides.^15–17^ Inspired by this, the guanidyl groups are modified on the TiO_2_, which will offer higher capacity of phosphopeptides binding due to the interaction between the phosphate groups and guanidyl groups.^18,19^

The aim of this work is to design and synthesize a novel MOAC material with guanidyl-functionalized TiO_2_ nanoparticles (GF-TiO_2_) anchored on the surface of graphene oxide (GO) platform (denoted as GF-TiO_2_–GO) for selective enrichment of phosphopeptides. The high specific surface area of graphene will offer higher capacity for loading GF-TiO_2_ and thus enhance the interactions between GF-TiO_2_ and phosphopeptides, which will result in a higher enrichment capacity to phosphopeptides. On the other hand, the introduction of guanidyl groups will also significantly enhance the capacity of phosphopeptides binding. The GF-TiO_2_–GO nanohybrids with the above properties are anticipated to have a highly selective enrichment ability toward phosphopeptides.

## 2. Experimental

**Fig. 1:**
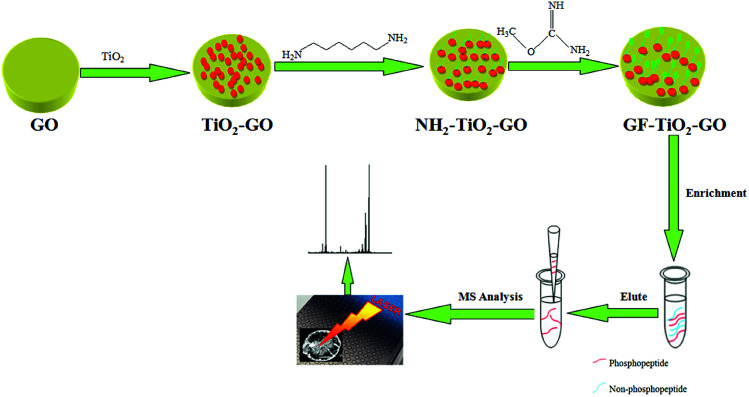
Schematic illustration for preparation of GF-TiO_2_–GO nanohybrids and the typical protocol for phosphopeptide enrichment.

As shown in Fig. 1, the TiO_2_ nanoparticles were synthesized through a sol–gel method in accordance with previous report.^20^ Graphene oxide (GO) was prepared from graphite powder (from Alfa Aesar) by a modified Hummers method and dispersed in water to form a homogeneous solution with a concentration of 0.5 mg mL^−1^. 20 mg of TiO_2_ particles were dispersed in aqueous solution of GO (30 mL) and stirred for 10 h at room temperature (RT) to obtain the TiO_2_ hybrid material (TiO_2_–GO). Then the obtained TiO_2_–GO nanohybrids were redispersed in aqueous solution of 1,6-hexanediamine (3 mg mL^−1^, 30 mL) and stirred at RT overnight to synthesize the 1,6-hexanediamine modified TiO_2_–GO nanohybrids (NH_2_–TiO_2_–GO). The NH_2_–TiO_2_–GO nanohybrids were collected and washed with water and then dispersed in aqueous solution of *O*-methylisourea hemisulfate (100 mg mL^−1^, 30 mL), the pH was adjusted to 11 by 1 M NaOH aqueous solution. The mixture was stirred at 60 °C for 8 h to obtain the GF-TiO_2_–GO nanohybrids. After washing by water several times, the obtained GF-TiO_2_–GO nanohybrids were dried under vacuum.

The enrichment performance of GF-TiO_2_–GO nanohybrids for phosphopeptides was investigated. A typical process is described as below: the tryptic protein digests were mixed with 0.3 mg GF-TiO_2_–GO nanohybrids in loading buffer (50% acetonitrile and 0.1% TFA water solution (v/v), 100 μL), and vibrated for 30 min and centrifuged to obtain the precipitates. Then, the obtained precipitates were rinsed with loading buffer (100 μL) three times. Finally, the phosphopeptides trapped by the GF-TiO_2_–GO nanohybrids were eluted using 10 μL 10% NH_3_·H_2_O under sonication for 10 min and the eluate was analyzed by matrix-assisted laser desorption/ionization time-of-flight mass spectrometry (MALDI-TOF MS). The human serum sample could be directly used for the enrichment of phosphopeptides by mixing the diluted human serum sample (100 μL) aqueous solutions and 0.3 mg GF-TiO_2_–GO nanohybrids and then treating the mixture in the procedure elucidated above for capturing the tryptic digestions of proteins.

## 3. Results and discussion

### 3.1. Characterization of GF-TiO_2_–GO

**Fig. 2:**
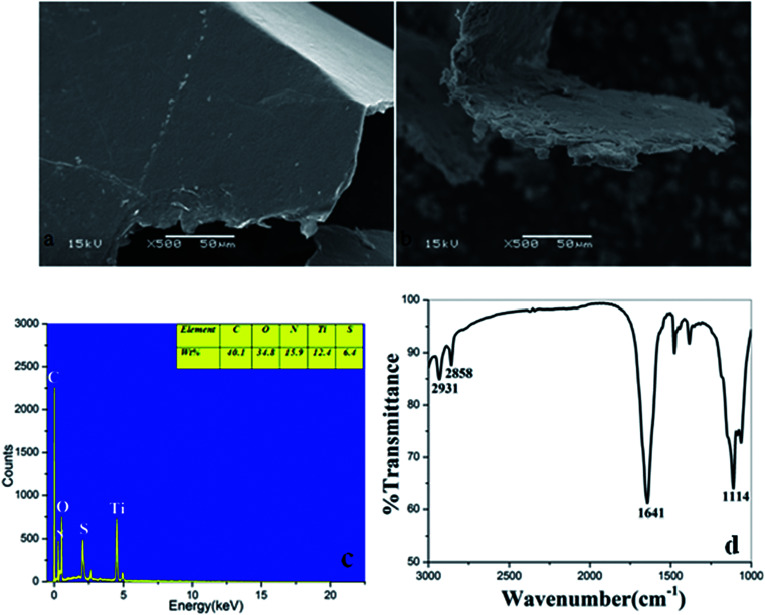
SEM images of (a) GO; (b) GF-TiO_2_–GO; (c) the spectra of EDX and (d) FTIR of GF-TiO_2_–GO.

The SEM images of GO and GF-TiO_2_–GO nanohybrids were shown in Fig. 2a and b. Obviously, GO exhibited a rather flat and clean surface. As a comparison, the GF-TiO_2_–GO nanohybrids had a much rougher surface (Fig. 2b), revealing that guanidyl-functionalized TiO_2_ nanoparticles had been assembled on the surface of GO with a robust layer. Moreover, the result of TEM images also indicated that the modified TiO_2_ nanoparticles (10–20 nm) has been anchored on the surface of GO (Fig. S1†). EDX spectrometry further showed that the GF-TiO_2_–GO nanohybrids were composed by Ti, C, N, O, S elements (Fig. 2c). The weight percentage was 15.9 wt% for N after C (40.1 wt%) and O (34.8 wt%), which indicated that amounts of N elements existed in the GF-TiO_2_–GO nanohybrids, further confirming the successful modification of TiO_2_ by the guanidyl groups.

The GF-TiO_2_–GO nanohybrids were also analyzed by IR spectroscopy. As shown in Fig. 2d, the characteristic absorbance peaks at 2931 cm^−1^ and 2858 cm^−1^ were assigned to C–H bonds on the alkyl chains of 1,6-hexanediamine moieties. Another, the strong absorption band at 1641 cm^−1^ was attributed to N–H bonds from the guanidyl groups. Also, the stretching vibration of C–N bonds from the guanidyl groups was observed at 1114 cm^−1^. As a comparision, the IR spectrum of TiO_2_ was also shown in Fig. S2,† the two peaks at 3425 cm^−1^ and 1351 cm^−1^ were assigned to the stretching and bending vibrations of the surface OH groups.^21,22^

### 3.2. Selectively enrichment of phosphopeptides from tryptic digestion of standard proteins

**Fig. 3:**
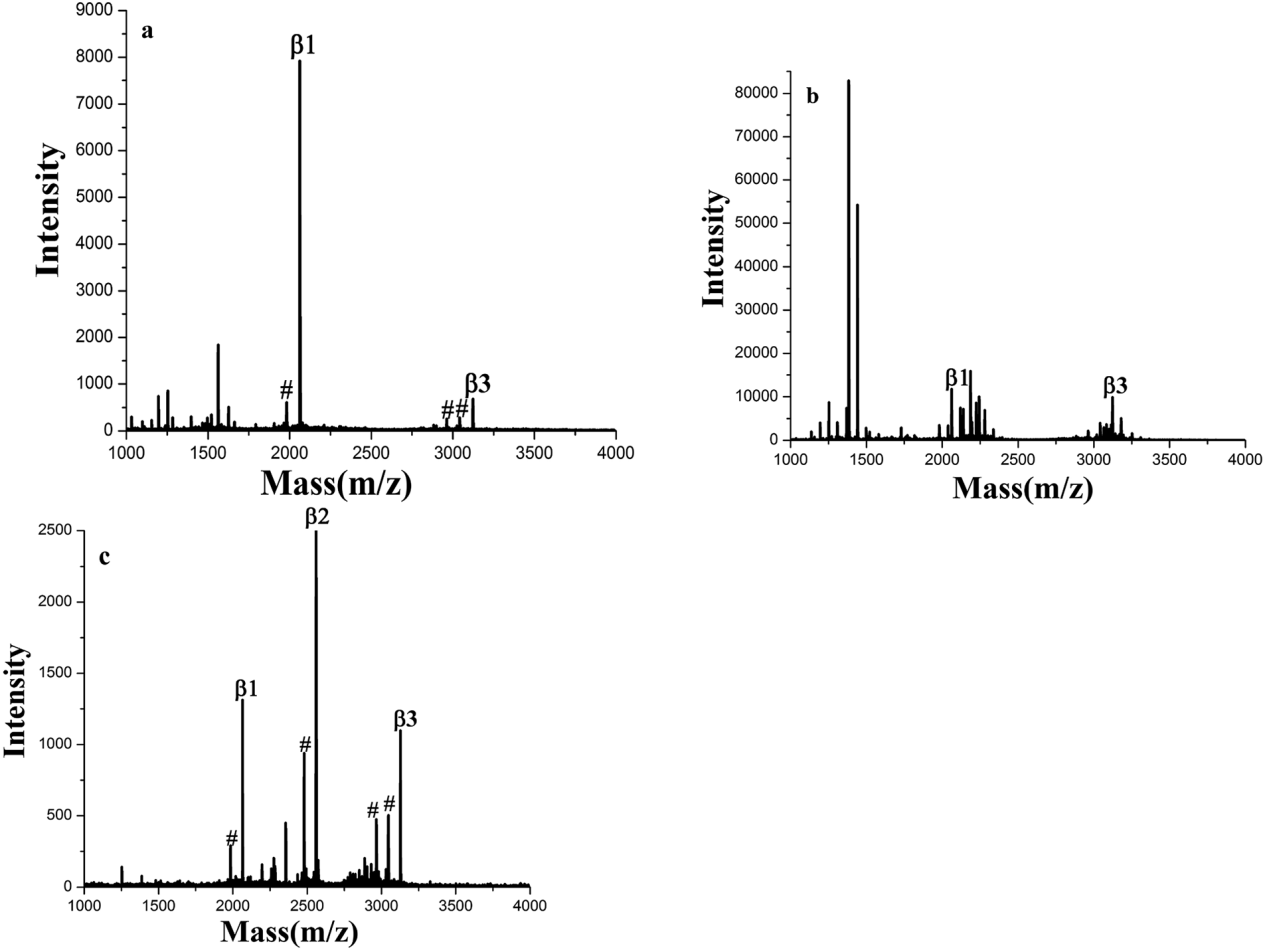
MALDI-TOF mass spectra of tryptic digests of β-casein: (a) direct analysis and after enriched by (b) GF-TiO_2_–GO and (c) TiO_2_ (# dephosphorylated fragment).

The enrichment capacity of GF-TiO_2_–GO nanohybrids toward phosphopeptides by MALDI-TOF MS was firstly validated. Direct analysis of β-casein digests (4 × 10^−6^ M) without enrichment only observed two phosphopeptides with poor signal/noise ratio (S/N), along with intensive signals of non-phosphopeptides (Fig. 3a). After enrichment by GF-TiO_2_–GO, the signals for the phosphopeptides significantly increased and dominated the spectrum with high S/N and clear background in Fig. 3b. As a comparison, the enrichment experiment was also carried out with the TiO_2_ nanoparticles. The mass spectrum showed only two phosphopeptides and some non-phosphopeptides with strong signals were observed (Fig. 3c). The results indicated that the GF-TiO_2_–GO nanohybrids had a better enrichment performance for the phosphopeptides. The detailed information of the observed phosphopeptides was listed in Table S1 (ESI†).

**Fig. 4:**
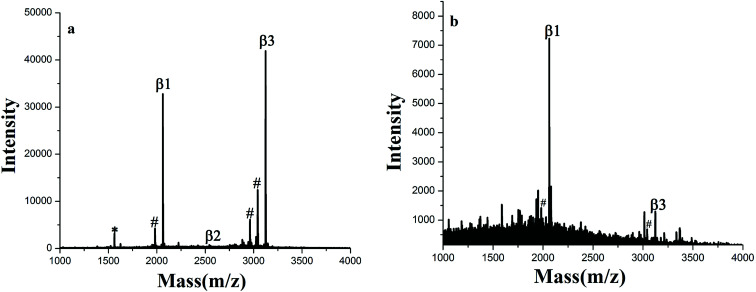
MALDI-TOF mass spectra of tryptic digests of (a) 5 × 10^−10^ M and (b) 1 × 10^−11^ M β-casein after enriched by GF-TiO_2_–GO (# dephosphorylated fragment, * doubly charged phosphopeptide).

The GF-TiO_2_–GO nanohybrids also had a high sensitivity for phosphopeptides. As shown in Fig. 4a, the signals from phosphopeptides in 5 × 10^−10^ M of β-casein digests could be easily detected. For the GF-TiO_2_–GO nanohybrids, two phosphopeptides could still be detected when the concentration of β-casein digests was as low as 1 × 10^−11^ M (Fig. 4b), while the signals were not detected when the concentration of phosphopeptides was 5 × 10^−10^ M for the TiO_2_ (Fig. S3†). The detect sensitivity was higher than our previous report (5 × 10^−11^ M) and some other reported enrichment materials.^23,24^ The improved enrichment capacity and higher detection sensitivity might be attributed to the introduction of GO and guanidyl groups. GO will expand the contact surface between GF-TiO_2_ and phosphopeptides, and thus enhance the interactions. The guanidyl groups modified on the surface of TiO_2_ will improve global phosphopeptides detection by the interactions between phosphate groups and amino groups.^25,26^

The MS signals in Fig. S4a and b† also suggested that the GF-TiO_2_–GO nanohybrids can be used as a stable enrichment material for continuous 20 days without signal reduction. To further confirm the reusability, the regenerated GF-TiO_2_–GO nanohybrids was reused to selectively capture β-casein digests for three times. As shown in Fig. S5a and b,† the result of enrichment in the third time is similar to that in the first time, indicating that GF-TiO_2_–GO nanohybrids could be reused for phosphopeptides enrichment. All these results further proved that the obtained GF-TiO_2_–GO nanohybrids had a remarkable performance in phosphopeptide enrichment.

**Fig. 5:**
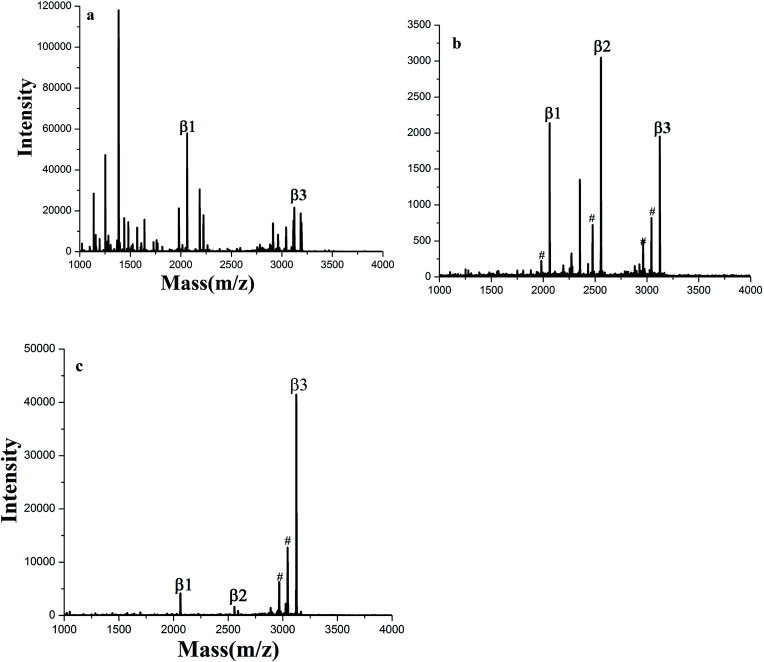
MALDI-TOF mass spectra of tryptic digests from a peptide mixture of β-casein (4 × 10^−6^ M) and BSA (4 × 10^−6^ M or 4 × 10^−4^ M): (a) direct analysis at a molar ratio of 1 : 1; after enriched by GF-TiO_2_–GO at molar ratios of (b) 1 : 1, (c) 1 : 100 (# dephosphorylated fragment).

The special selectivity of the GF-TiO_2_–GO nanohybrids toward phosphopeptides was also investigated using a more complex tryptic digests of β-casein and BSA mixture at a molar ratio 1 : 1 and 1 : 100 as a mimic biological sample for test. As shown in Fig. 5a, without enrichment, nonphosphopeptides peaks dominated nearly the whole spectrum, and there were only two phosphopeptides observed. However, after enrichment by GF-TiO_2_–GO, there were no nonphosphopeptides peaks, and the peaks from phosphopeptides were clearly detected (Fig. 5b). Even when the molar ratio of β-casein and BSA was decreased to 1 : 100, three phosphopeptides could be still be detected with high S/N (Fig. 5c). For TiO_2_, when the tryptic digests of β-casein and BSA were at a molar ratio 1 : 1, two phosphopeptides with background interference ions were observed, while no phosphopeptides were detected using the digests mixture of 1 : 100 (Fig. S6a and b†). Comparing with TiO_2_, the results further demonstrated that the GF-TiO_2_–GO nanohybrids had an excellent performance in selective capture of phosphopeptides from the complex peptide mixture.

### 3.3. Highly specific selective enrichment of phosphopeptides from human serum and nonfat milk

**Fig. 6:**
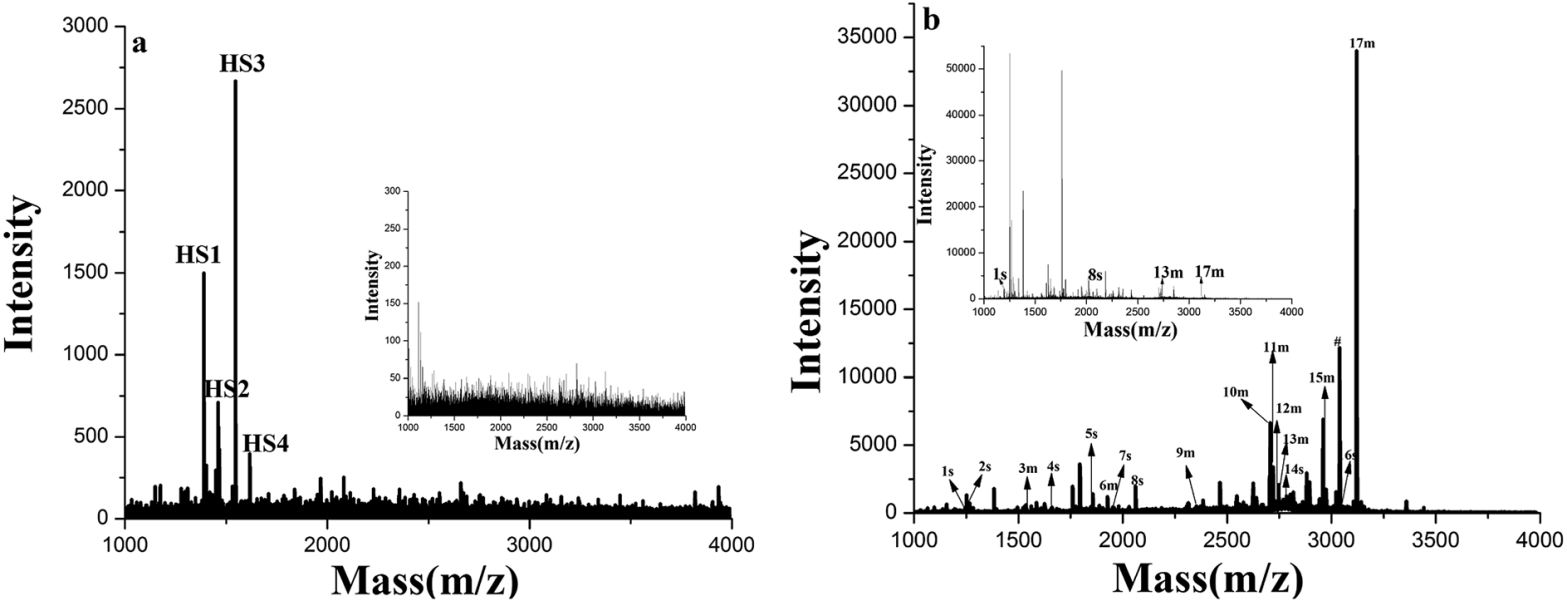
(a) MALDI-TOF mass spectra of human serum before (insetted graph) and after enriched by GF-TiO_2_–GO; (b) MALDI-TOF mas spectra of tryptic digests of nonfat milk before (insetted graph) and after enriched by GF-TiO_2_–GO (# dephosphorylated fragment).

To further test the application of GF-TiO_2_–GO nanohybrids toward phosphopeptides enrichment, the human serum and a tryptic digest of nonfat milk were chosen as a real biological sample to validate the enrichment performance.^27^ As shown in Fig. 6a, before enrichment, no phosphopeptides were detected from the diluted human sample. In contrast, four endogenous phosphopeptides derived from phosphorylated fibrinopeptide A in the human serum could be detected with high S/N after enrichment (the detailed sequence information of four endogenous phosphopeptides in the human serum was listed Table S2, ESI†). Similar results were also observed in the direct analysis of the tryptic digests of nonfat milk, in which nonphosphopeptides dominated the spectrum and only 4 weak peaks were generated from phosphopeptides. However, 17 phosphopeptides were identified after enrichment with GF-TiO_2_–GO (Fig. 6b). (The detailed sequence information of 17 phosphopeptides in the tryptic digest of nonfat milk was listed Table S3, ESI†). These results further demonstrated that the GF-TiO_2_–GO nanohybrids were capable of selectively capturing phosphopeptides from the extremely complicated biological samples.

## 4. Conclusion

A novel TiO_2_-based MOAC hybrid nanomaterial, GF-TiO_2_–GO, was successfully synthesized by guanidyl-functionalized TiO_2_ nanoparticles anchored on the surface of graphene oxide platform for the selective enrichment of phosphopeptides. GF-TiO_2_–GO exhibited large capacity of phosphopeptides enrichment and improved selectivity to phosphopeptides and showed a higher enrichment efficiency toward phosphopeptides for the complicated biological samples. This work provided an approach to prepare more efficient TiO_2_-based MOAC hybrid nanomaterials for phosphoproteome research.

## Conflicts of interest

There are no conflicts to declare.

## Supplementary Material

RA-008-C8RA05006F-s001
