# Correction: A guanidyl-functionalized TiO_2_ nanoparticle-anchored graphene nanohybrid for enhanced capture of phosphopeptides

**DOI:** 10.1039/c8ra90074d

**Published:** 2018-09-13

**Authors:** Hailong Liu, Bin Lian

**Affiliations:** College of Life Sciences, Nanjing Normal University No. 1, WenYuan Road QiXia District Nanjing 210023 Jiangsu Province China bin2368@vip.163.com; State Key Laboratory of Environmental Geochemistry, Institute of Geochemistry, Chinese Academy of Sciences Guiyang China

## Abstract

Correction for ‘A guanidyl-functionalized TiO_2_ nanoparticle-anchored graphene nanohybrid for enhanced capture of phosphopeptides’ by Hailong Liu *et al.*, *RSC Adv.*, 2018, **8**, 29476–29481.

The authors regret that there was an error in Fig. 3 of the original article, as the three parts of the figure were labelled incorrectly. The correct version of Fig. 3 is presented below.

**Fig. 1:**
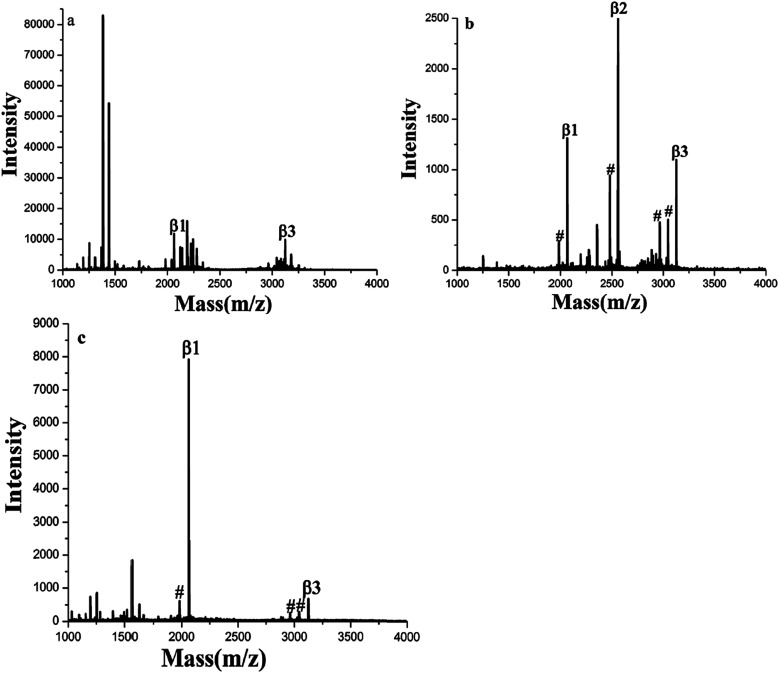
MALDI-TOF mass spectra of tryptic digests of β-casein: (a) direct analysis and after enriched by (b) GF-TiO_2_–GO and (c) TiO_2_ (# dephosphorylated fragment).

The Royal Society of Chemistry apologises for these errors and any consequent inconvenience to authors and readers.

## Supplementary Material

