# Supplementary material for: A guanidyl-functionalized TiO2 nanoparticle-anchored graphene nanohybrid for enhanced capture of phosphopeptides
Source: RSC Adv. 2018 Aug 20;8(51):29476–81. doi: 10.1039/c8ra05006f (PMC9084561; doi:10.1039/c8ra05006f)
Supplement: RA-008-C8RA05006F-s001 [file RA-008-C8RA05006F-s001.pdf]

Electronic Supplementary Material (ESI) for RSC Advances.
This journal is © The Royal Society of Chemistry 2018

Supporting Information

A guanidyl-functionalized TiO2 nanoparticle-anchored graphene nanohybrid for enhanced capture of phosphopeptides

Hailong Liu[1,2], Bin Lian[1*]

[1] College of Life Sciences, Nanjing Normal University, Nanjing, 210023, China

[2] State Key Laboratory of Environmental Geochemistry, Institute of Geochemistry, Chinese Academy of Sciences, Guiyang, China

Experimental details

Materials and chemicals

Trifluoroacetic acid (TFA), β-casein, bovine serum albumin(BSA), ammonium bicarbonate(NH_4HCO_3) , O-methylisourea hemisulfate and 2,5-dihydroxybenzoic acid (2,5-DHB) were purchased from Sigma-Aldrich (St. Louis, MO, USA). Sequencing grade modified trypsin was obtained from Promega (Madison, USA). Acetonitrile (ACN) was obtained from Merck (Darmstadt, Germany). Graphite powder (99.9995%, 325 mesh) was from Alfa Aesar. Human serum was supplied by Jiangsu Province Hospital of TCM. Nonfat milk was purchased from a local supermarket. All aqueous solutions were prepared using Milli-Q water by Milli-Q purification system (Millipore, Milford, MA, USA).

Sample preparation

β-casein and bovine serum albumin (BSA) were dissolved in 25 mM ammonium bicarbonate buffer solution (pH=8.1) containing trypsin at the ratio of enzyme-to-protein of 1:40 (w/w) at 37 °C for 16 h. Human serum was diluted to five times with 50% acetonitrile and 0.1% TFA aqueous solution (v/v). 50 μL Nonfat milk was diluted to 250 μL in 50 mM ammonium bicarbonate buffer solution. After centrifugation at 14000 rpm for 25 min, the supernatant was denatured at 100 ℃ for 5 min, 10 μg trypsin was added and then the mixture was incubated for 12 h at 37 ℃.

Characterization and measurements

The Infrared spectra were obtained from a Nicolet NEXUS 670 Fourier transform infrared spectrometer. The Scanning electron microscopy (SEM) images were determined by a JSM-5610LV scanning electron microscope system. Transmission electron microscopy (TEM) images were made on a JEOL JEM-2100F transmission electron microscopy operated at an accelerating

voltage of 200 kV. The chemical composition of the GF-TiO_2-GO nanohybrids was obtained by a Bruker QX200 Energy-dispersive X-ray(EDX) spectrometer.

MALDI-TOF MS analysis

For MALDI-TOF MS, 1μL of the eluate was deposited on a MALDI plate and dried in the air, and then 1μL of 2,5-DHB (20 mg/mL, dissolved in 50% ACN (v/v) containing 1% H_3PO_4) was deposited and dried. All the MALDI TOF MS spectra were recorded with MALDI TOF/TOF MS (BRUKER Daltonics, Germany) in reflected positive-ion mode with 200 laser shots in each spectrum.

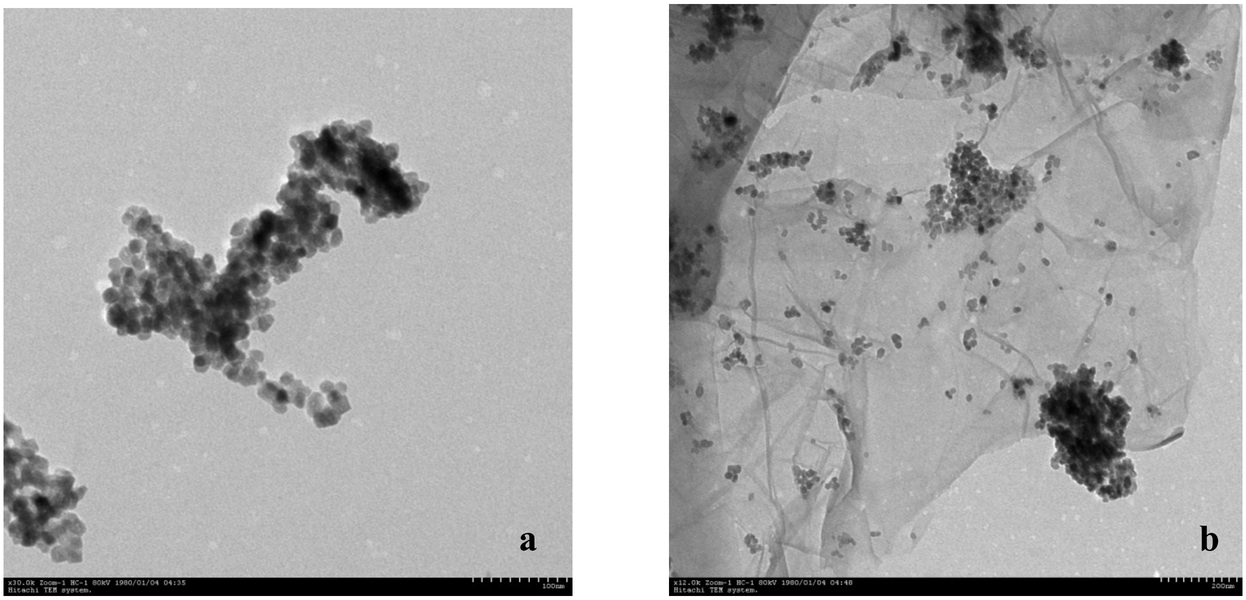

Fig. S1. TEM images of (a) TiO_2; (b) GF-TiO_2-GO

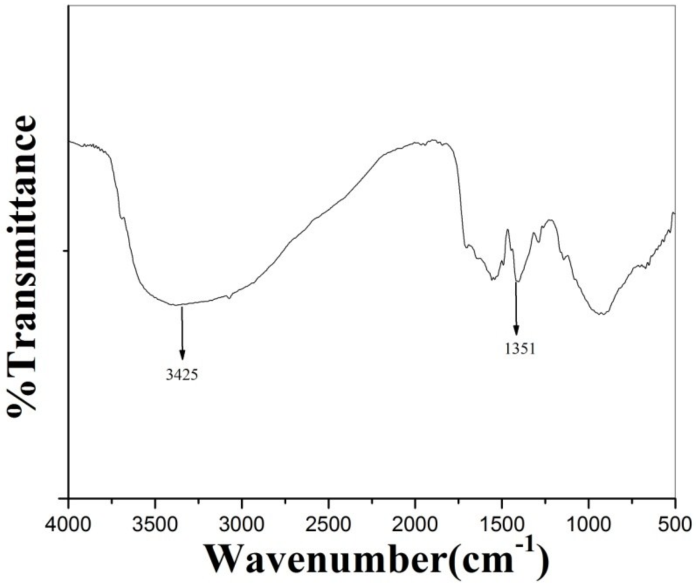

Fig. S2. FTIR spectra of TiO_2.

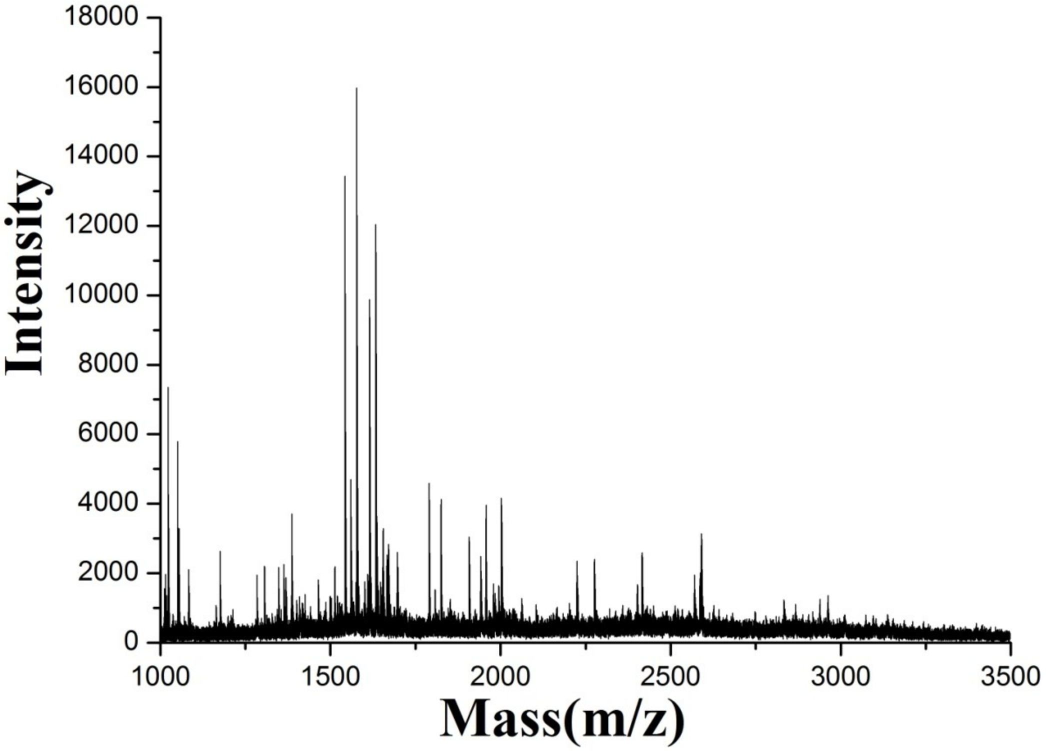

Fig. S3. MALDI-TOF mass spectra of tryptic digests of 5×10^{-10}M β-casein after enriched by TiO_2.

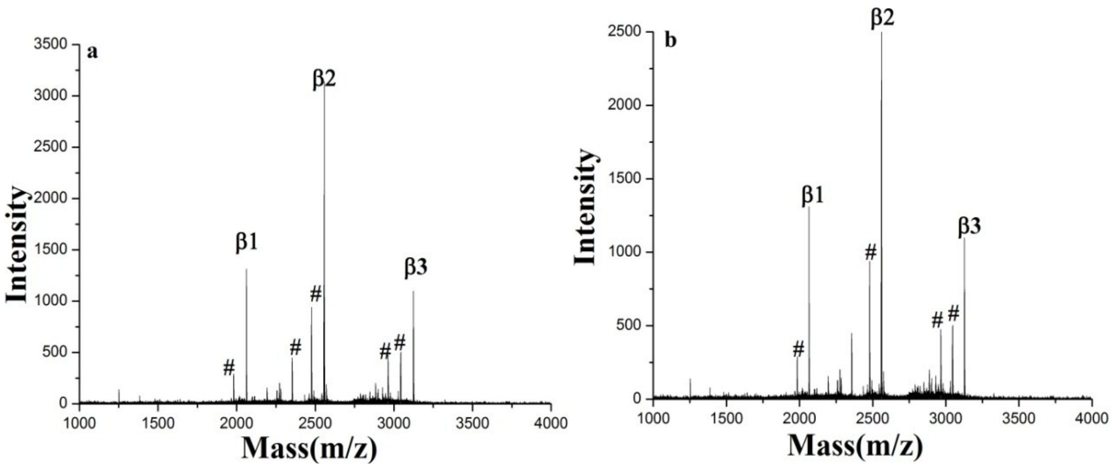

Fig. S4. MALDI-TOF mass spectra of tryptic digests from a peptide mixture of β-casein after enriched by GF-TiO_2-GO after 1 day(a) and 20 days (b). (# dephosphorylated fragment)

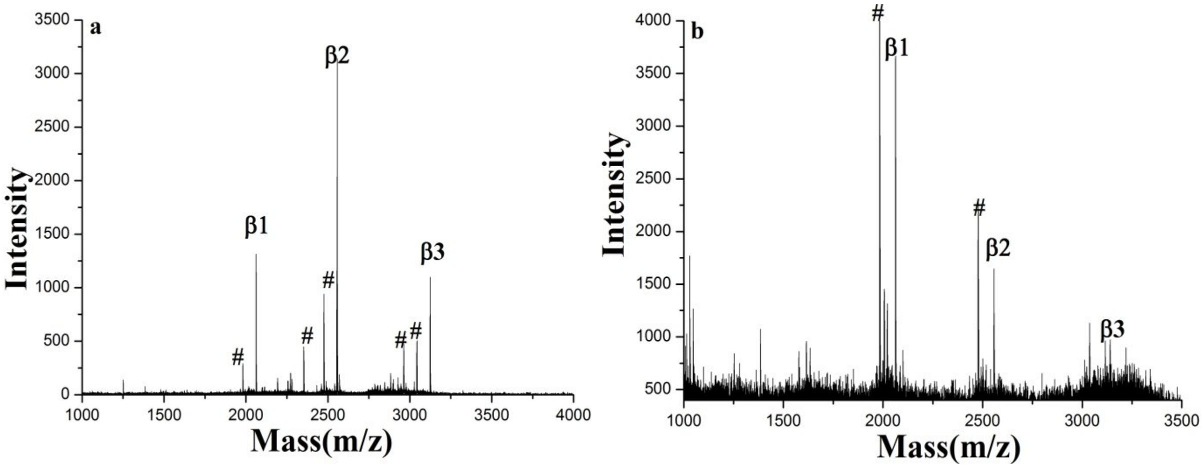

Fig. S5. MALDI-TOF mass spectra of tryptic digests from a peptide mixture of β-casein after enriched by GF-TiO_2-GO for the first time(a) and the third time (b). (# dephosphorylated fragment)

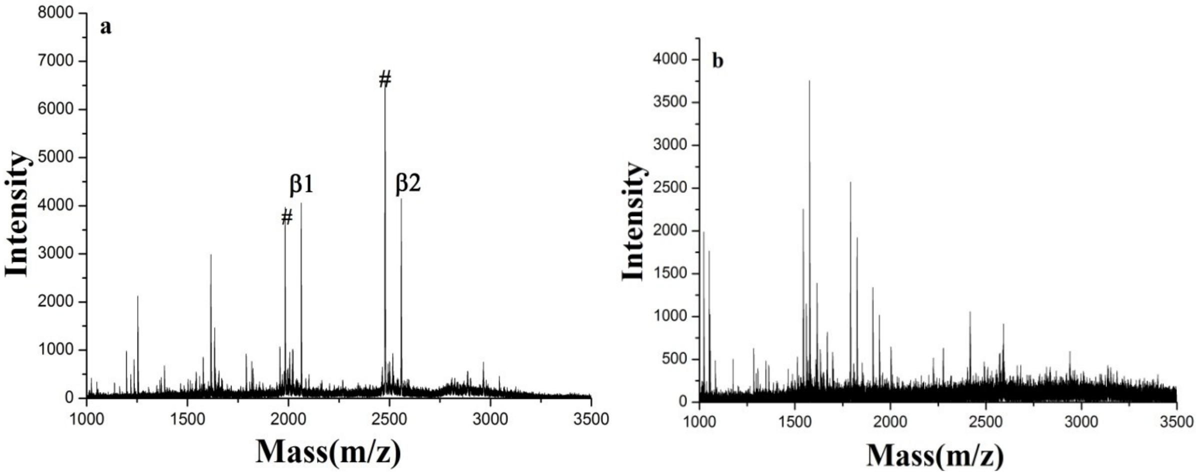

Fig. S6. MALDI-TOF mass spectra of tryptic digests from a peptide mixture of β-casein (4×10^{-6}M) and BSA(4×10^{-6}M or 4×10^{-4}M): (a) direct analysisat at a molar ratio of 1:1; after enriched byTiO_2 at molar ratios of (b) 1:1, (c) 1:100. (# dephosphorylated fragment)

NO.	Peptide sequence	Number of phosphoryl groups	Observed m/z
β1	FQ[pS]EEQQQTEDELQDK	1	2061.72
β2	FQ[pS]EEQQQTEDELQDKIHPF	1	2556.09
β3	RELEELNVPGEIVE[pS]L[pS][pS][pS]EESITR	4	3122.27

[pS]: phosphorylated site.

Table S1 The detailed information for the detected phosphopeptides from tryptic digests of β -casein after enriched by GF-TiO_2-GO in MALDI-TOF MS analysis

NO.	Peptide sequence	Number of phosphoryl groups	Observed m/z
HS1	D[pS]GEGDFLAEGGGV	1	1389.46
HS2	AD[pS]GEGDFLAEGGGV	1	1460.61
HS3	D[pS]GEGDFLAEGGGVR	1	1545.58
HS4	AD[pS]GEGDFLAEGGGVR	1	1616.71

[pS]: phosphorylated site.

Table S2 The detailed information for the detected phosphopeptides from human serum sample after enriched by GF-TiO_2-GO in MALDI-TOF MS analysis

NO.	Peptide sequence	Number of phosphoryl groups	Observed m/z
1s	TVDMESTEVF	1	1237.6
2s	TVDMMESTEVF	1	1254.6
3m	EQLSTSEENSKK	2	1539. 6
4s	VPQLEIVPNSAEER	1	1660.8
5 s	DIGSESTEDQAMEDIK	1	1847.0
6 m	DIGSESTEDQAMEDIK	2	1927.7
7s	YKVPQLEIVPNSAEER	1	1952.0
8 s	FQSEEQQQTEDELQDK	1	2061.7
9 m	NVPGEIVESLSSSEESITR	4	2352.9
10 m	QMEAESISSSEEIVPNSVEAQK	5	2704.4
11 m	QMEAESISSSEEIVPNSVEQK	5	2720.4
12m	QoMEAESISSSEEIVPNSVEQK	5	2735.3
13 m	NTMEHVSSSEESIISQETYKQ	4	2747.4
14s	KIEKFQSEEQQQTEDELQDKIHPF	1	2779.7
15m	ELEELNVPGEIVESLSSSEESITR	4	2965.9
16s	KIEK FQSEEQQQTEDELQDKIHPF	1	3054.1
17 m	RELEELNVPGEIVESLSSSEESITR	4	3122.3

s: single phosphopeptide; m: multiple phophopeptide; o: oxidation on methionine

#: metastable losses of phosphoric acid

Table S3 The detailed information of the observed phosphopeptides obtained from tryptic digests of non-fat milk.
